# Platelet-rich plasma: A promising therapy for mitigating sperm oxidative stress and mitochondrial dysfunction in subfertile men

**DOI:** 10.1371/journal.pone.0319471

**Published:** 2025-04-28

**Authors:** Rim Kooli, Manel Boussabbeh, Dhekra Chebil, Abderraouf Kenani, Linda Khefacha, Meriem Mehdi, Amira Sallem

**Affiliations:** 1 Department of Reproductive Biology, Maternity and Neonatology Center, Fattouma Bourguiba University Teaching Hospital, Monastir, Tunisia; 2 Laboratory of Research on Biologically Compatible Substances, Faculty of Dentistry of Monastir, Monastir University, Monastir, Tunisia; 3 Faculty of Medicine of Sousse, University of Sousse, Sousse, Tunisia; 4 Research Laboratory “Environment, Inflammation, Signaling and Pathologies” (LR18ES40), Faculty of Medicine of Monastir, University of Monastir, Monastir, Tunisia; 5 Department of Biology, Maternity and Neonatal Medicine Center, Fattouma Bourguiba University Hospital, Monastir, Tunisia; 6 Laboratory of Histology-Embryology and Cytogenetics (LR 18 ES 40), Faculty of Medicine of Monastir, University of Monastir, Monastir, Tunisia; Ladoke Akintola University of Technology, NIGERIA

## Abstract

Platelet-rich plasma (PRP) is a pioneering therapy widely used in various medical fields, showing promising outcomes. However, its impact on human sperm quality remains poorly explored among emerging therapies. This study aims to investigate the effect of autologous PRP supplementation on oxidative stress levels and mitochondrial activity in human sperm. PRP was freshly prepared from venous blood and added to each ejaculated semen sample at different concentrations of 2%, 5%, and 10%. Reactive oxygen species (ROS) in spermatozoa were measured after 24 hours of incubation at 37° (5% CO2), using nitro-blue tetrazolium (NBT) test. The MTT test was used to measure the mitochondrial succinate deshydrogenase activity. A total of 180 semen samples were obtained from 15 patients. The supplementation with PRP significantly reduced the reactive oxidative species levels and improved mitochondrial activity in spermatozoa. The level of oxidative stress in sperm was significantly decreased after 24h of incubation with PRP at 2% (p = 0.001), 5% (p = 0.001) and 10% (p = 0.001) when compared to the control group. The succinate dehydrogenase activity was enhanced in the three groups when compared to the control group. It increased from 0.667 ± 0.313 to 0.952 ± 0.499 (p = 0.018), 1.201 ± 0.657 (p = 0.002) and 1.159 ± 0.607 (p = 0.001) after incubation with 2%, 5% and 10% of PRP, respectively. This study has shown that PRP supplementation could be a promising tool to enhance sperm quality against oxidative stress and mitochondrial dysfunction. These findings could be a starting point to investigate the usefulness of PRP in ART procedures.

## Introduction

Infertility is a widespread concern impacting approximately 70 million people worldwide. According to the World Health Organization, around 9% of couples struggle with fertility issues with male factor contributing to 50% of these cases [1]. Male infertility arises from various origins, including genetic mutations, lifestyle factors, medical and surgical conditions or medication. Despite tremendous advancements in investigations, male infertility remains idiopathic in 30% of cases [1].

Semen oxidative stress (OS), defined by the imbalance between reactive oxygen species and semen’s antioxidant capacity, stands as a primary contributor to infertility, impacting 30% to 80% of infertile men [2]. Indeed, sperm and seminal leukocytes are the main sources of the overproduction of ROS leading to male infertility by two key mechanisms: (i) damaging the sperm membrane which affects sperm motility and hence spermatozoa ability to fuse with the oocyte. (ii) ROS can alter the sperm DNA integrity resulting in defective paternal genetic patrimony.

Given the key role of oxidative stress in impairing sperm quality, the heightened attention towards antioxidants has emerged. Common antioxidants such as vitamin E, vitamin C, and coenzyme Q10 exhibit efficacy in decreasing oxidative stress and improving sperm parameters.

Platelet-rich plasma (PRP) is an autologous blood-derived product that contains platelet concentrations at least 2 to 3 times above the normal level and includes platelet-related growth factors [3]. PRP emerges as a novel therapeutic option that is being used in multiple medical fields [3–5]. Indications for PRP therapy span from addressing muscle and skeletal injuries to promoting hair re-growth, demonstrating promising outcomes in dermatology, orthopedics, and dentistry. Recent emerging evidence highlights its positive effects in reproductive medicine [6–9]. The first trial on the use of PRP in human reproduction technologies was reported by a Chinese group to improve endometrial thickness in patients undergoing IVF treatment [10]. When focusing on the usefulness of PRP in managing infertility, much more studies were interested in investigating its impact on the female partner than the male.

Due to its high efficacy and its richness in growth factors, PRP may offer an interesting therapeutic option in male infertility. Hence, this study aims to assess the effect of PRP supplementation on human sperm quality, focusing on sperm oxidative stress levels and mitochondrial activity.

## Materials and methods

### 1. Study population

We included in the current study patients addressed to the Department of Reproductive Biology of Fattouma Bourguiba University teaching Hospital (Monastir, Tunisia) for semen quality assessment from 01 May 2023 to 30 October 2023 and having a platelets counting >  150 000 UI.

Participants with a semen volume less than 1 ml or a sperm concentration less than 1x10⁶ cells/mL were not included in the study. All patients suffering from andrological disorders, inflammatory and immune diseases, or recent fever, or undertaking any treatment that may alter spermatogenesis were excluded from the study.

### 2. Semen collection

Semen samples, obtained by masturbation and collected into sterile containers, were incubated at 37 °C for 30 min to allow liquefaction. Each sample was then evaluated for sperm concentration, motility, morphology, and viability.

Semen parameters were interpreted according to the World Health Organization manual for the examination of human semen (WHO 2021).

### 3. Autologous PRP preparation

The PRP preparation procedure consisted of two centrifugation steps. The initial centrifugation at 300 g for 10 minutes at room temperature separates the whole blood into three layers: an upper layer containing mostly plasma and white blood cells (WBC), an intermediate thin layer known as the platelet rich plasma (PRP), and a bottom layer consisting mostly of red blood cells (RBC). The RBC layer is eliminated and the WBC and PRP are transferred to an empty sterile tube and centrifuged at 600 g for 10 minutes. After the second centrifugation, the PRP will settle in the bottom of the tube and the supernatant is eliminated. After collection of the PRP, platelets are activated with CaCl2 (10M).

### 4. Sperm treatment

Each semen sample was divided into four aliquots and incubated with increasing concentrations of PRP (0%, 2%, 5% and 10%) for 24 hours at 37°C and 5% CO2. The following measurements of ROS amounts and mitochondrial activity were blindly performed (control versus different PRP concentrations).

### 5. Measurement of intracellular reactive oxygen species (NBT test)

Following the 24 hours of incubation, semen samples were washed with PBS then resuspended in 200 μL of PBS. Triplicate samples of 100μL of washed semen were incubated with an equal volume of NBT working reagent (1:10 diluted in PBS from 0.01% NBT stock; Sigma-Aldrich, St Louis, MO, USA) at 37°C for 45 min. Samples were then washed and centrifuged at 500 g for 10 minutes in PBS twice to remove all residual NBT solution, leaving only a cell pellet containing formazan. To be quantified, the intracellular formazan was solubilized in 60μL of KOH (2M) and dimethyl sulphoxide (DMSO) (Sigma-Aldrich) and the resulting color reaction was measured spectrophotometrically on a microplate reader (Model ELx800; Bio-Tek Instruments, Inc., Winooski, VT, USA) at 630 nm [11,12].

### 6. Measurement of mitochondrial activity (MTT test)

The MTT assay, a method based on the reduction of the tetrazolium salt (3-[4,5-dimethylthiazol-2-yl]-2,5-diphenyltetrazolium bromide), offers a sensitive mean to assess cellular metabolic activity, especially in mitochondria. After PRP treatment, spermatozoa were exposed to MTT solution (final concentration of 0.5 mg/ml) for 1 hour [13]. Subsequently, the formed dark-blue formazan crystals in spermatozoa were dissolved using DMSO, and their absorbance at 570 nm was quantified utilizing a spectrophotometer microplate reader (Bioteck, Elx 800). The findings were presented as the absorbance of MTT reduction.

### 7. Ethical approval

The current study was conducted in adherence to ethical principles in research. We obtained approval from the Ethics Committee of the Faculty of Medicine in Monastir under the number “IORG 0009738 N°115/OMB 0990-0279”.

### 8. Statistical analysis

Data analyses were performed using Statistical Package for Social Sciences for Windows (SPSS) version 26.

Baseline clinical characteristics and semen parameters were expressed as mean ±  SD or median [interquartile range (IQR)] as appropriate for continuous numerical variables and as frequency (percentage) for categorical variables. Between-groups comparisons according to PRP concentration were performed with Wilcoxon test.

## Results

### 1. Description of the study population

A total of 180 semen samples from 15 patients were collected in the current study. The median age of the study population was of 36 [34–40] years old. Sperm characteristics of the study population are detailed in Table 1.

**Table 1:** Semen characteristics in the study population.

Variable	Median	IQR * [25% - 75%]
**Volume (ml)**	3	2,2-3
**Sperm Concentration (million/ml)**	90	62-240
**Progressive Motility (%)**	20	15-25
**Total Motility (%)**	37	30-41,25
**Abnormal Morphology (%)**	94	91-96

### 2. Sperm reactive oxygen species: NBT assay

The basic level of oxidative stress as assessed by formazan absorbance in the included semen samples was of 0.373 ± 0.25 and significantly decreased after 24h of incubation with PRP at the three studied concentrations. A drop of respectively about 1.56 fold (0.239 ± 0.13), 1.75 fold (0.21 ± 0.10) and 1.64 fold (0.22 ± 0.15) was seen at 2% PRP (p = 0.001), 5% PRP (p = 0.001) and 10% PRP (p = 0.001) respectively when compared to the control group.

The best improvement in oxidative stress status (38%) was observed at 5% PRP.

**Fig 1:**
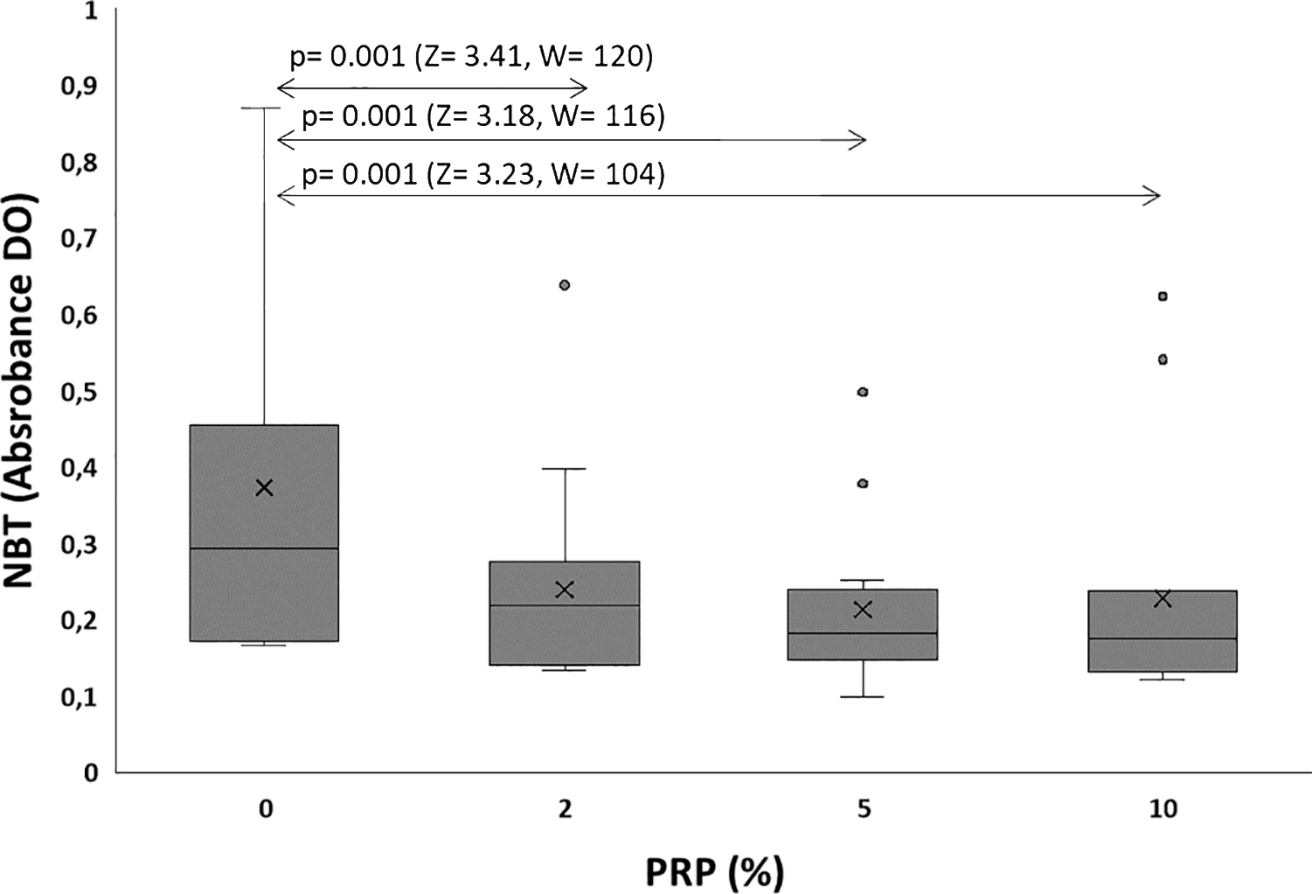
Effect of PRP on spermatozoa ROS production as assessed by the NBT test. Z: The Z-score or standardized test statistic and W: The Wilcoxon test statistic.

**Fig 2:**
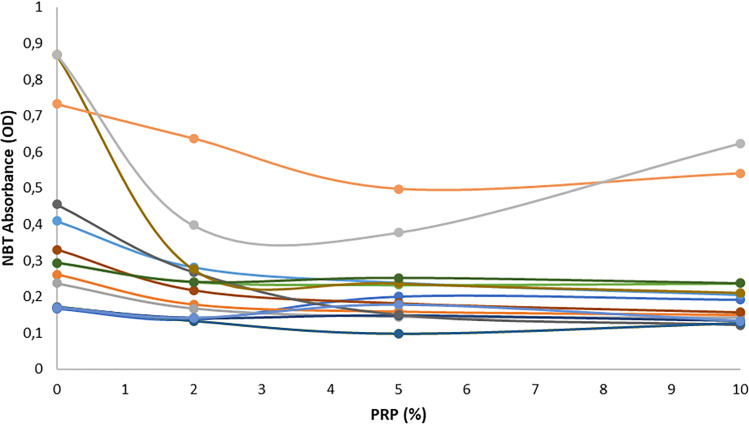
Line chart of NBT absorbance after PRP supplementation. PRP at 0%, 2%, 5% and 10% was supplemented in each semen sample.

Results related to ROS levels as revealed by NBT assay in the entire population are presented in Fig 1 and line chart of each patient are illustrated in Fig 2.

### 3. Sperm mitochondrial activity: MTT assay

**Fig 3:**
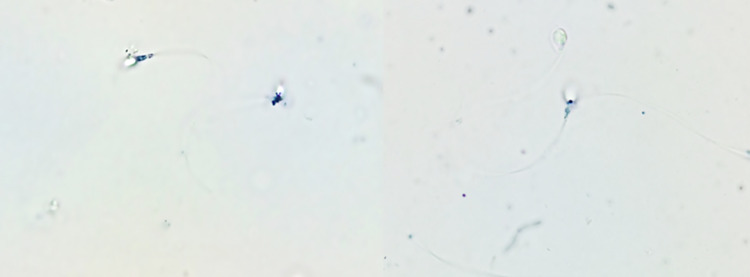
Localization of MTT reduction (formazan) in human spermatozoa.

We began by checking the localization of MTT reduction in spermatozoa to demonstrate that this assay is the mirror of mitochondrial activity in male gamete. As demonstrated in Fig 3, formazan is only located in the midpiece of the spermatozoon which corresponds to the mitochondrial activity.

The succinate dehydrogenase activity of the spermatozoa was ameliorated in the three treated groups when compared to the control group. As measured by the MTT test, the absorbance of formazan increased about 1.42-fold from 0.667 ± 0.313 to 0.952 ± 0.499 (p = 0.018) at 2% PRP, and about 1.8 fold to reach 1.201 ± 0.657 (p = 0.002) at 5% and about 1.73 fold (1.159 ± 0.607; p = 0.001) after 10% PRP incubation.

The best enhancement in succinate deshydrogenase activity (80%) was seen with 5% PRP.

**Fig 4:**
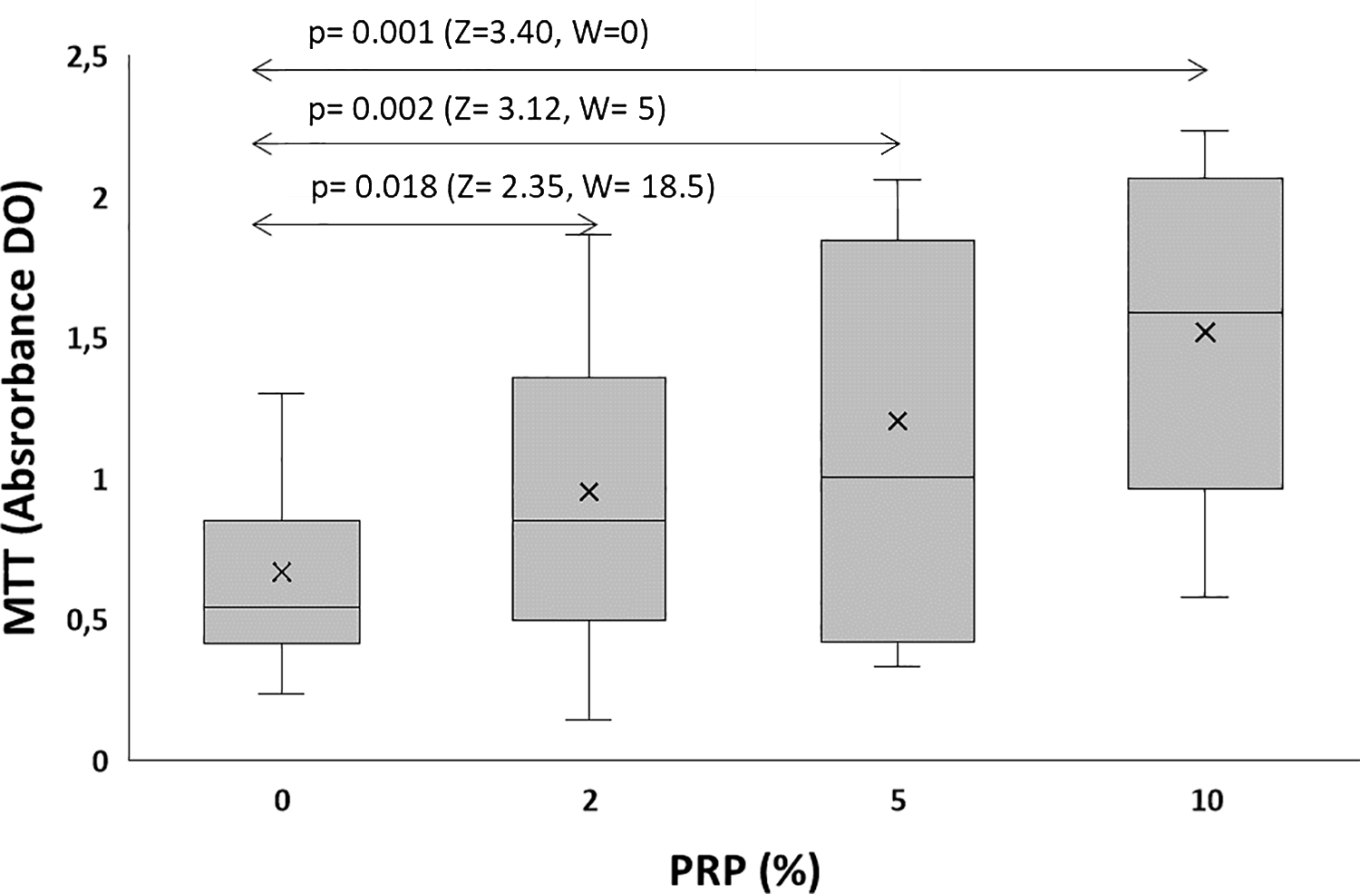
Effect of PRP on succinate dehydrogenase activity as assessed by the MTT test. Z: The Z-score or standardized test statistic and W: The Wilcoxon test statistic.

**Fig 5:**
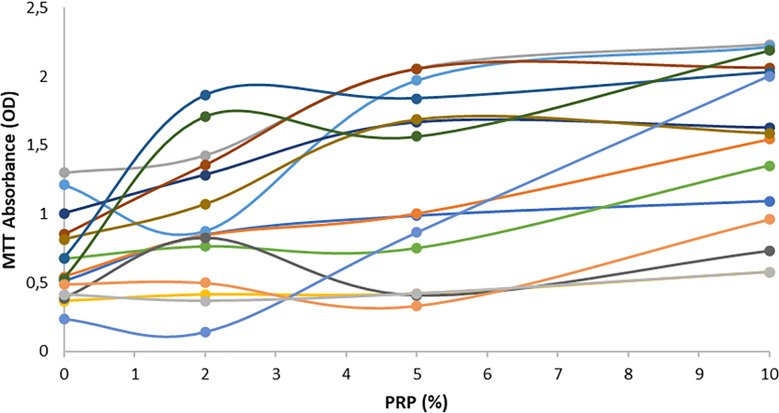
Line chart of MTT absorbance after PRP supplementation. PRP at 0%, 2%, 5% and 10% was supplemented in each semen sample.

Fig 4 shows succinate dehydrogenase activity following semen PRP incubation and Fig 5 illustrates line chart of each patient.

## Discussion

We have shown through the current study the usefulness of autologous PRP in reducing seminal oxidative stress and enhancing succinate dehydrogenase activity mainly at 5% concentration.

Platelet-Rich Plasma (PRP) therapy has shown promising outcomes in the reproductive field, notably concerning ovarian and endometrial infertility related factors [10,14,15]. However, there has been limited exploration into its effects on human male infertility. Indeed, from the first publication in 2019 to date, only three studies [16–18] have investigated the effect of PRP on human semen and only one of them has focused on fresh semen samples [18]. This underlies the importance of the current study which investigated the impact of *in vitro* supplementing of fresh semen with PRP on oxidative stress and mitochondrial succinate dehydrogenase (SDH) activity. By focusing on these parameters, we sought to elucidate the potential benefits of PRP in enhancing sperm quality, thereby contributing to a more comprehensive understanding of its therapeutic applications in male reproductive health.

Spermatozoa are particularly vulnerable to oxidative damage due to not only their limited antioxidant capacity but also their high content of polyunsaturated fatty acids in their membranes, making them vulnerable to lipid peroxidation [19]. This vulnerability is significant given that reactive oxygen species (ROS) play a dual role in male reproductive physiology. While physiological levels of ROS are essential for processes such as sperm capacitation and the acrosome reaction, excessive ROS production can lead to detrimental effects on sperm quality, ultimately contributing to male infertility [20].

Lines of evidence has shown that elevated ROS levels can induce lipid peroxidation, resulting in compromised sperm membrane integrity, decreased motility, and increased DNA fragmentation [21]. Epidemiological studies indicate that high levels of ROS are found in 30-80% of infertile men, highlighting their critical role in male reproductive health problems [2]. Factors such as lifestyle choices, environmental stressors, and underlying health conditions can exacerbate oxidative stress, further complicating male infertility. Current management strategies often focus on lifestyle modifications and antioxidant therapies to mitigate oxidative stress and improve sperm quality, although there remains a lack of consensus on optimal testing and treatment protocols for ROS-related infertility [20,22,23].

In the current study, we demonstrated a significant decrease in spermatozoa oxidative stress levels after 24 hours of PRP treatment with a best reduction of 38% (p = 0.001) at 5% of PRP. These findings are in line with a recent study by Bader et al., who demonstrated that autologous PRP improve sperm motility and decrease ROS overproduction induced by H_2_O_2_ [18]. Similarly, Nabavinia et al. showed that PRP supplementation during cryopreservation, known as a source of ROS overproduction, preserved sperm motility, viability, and plasma membrane integrity [16]. These findings collectively support the potential of PRP in mitigating oxidative stress and ameliorating sperm quality.

The observed decrease in oxidative stress levels upon PRP treatment could be explained by the presence of antioxidant Zn/Cu SOD enzyme in the platelet rich plasma [24]. Indeed, Zn/Cu SOD catalyzes the conversion of superoxide radicals into hydrogen peroxide and oxygen, reducing ROS levels and mitigating oxidative stress in the extracellular environment. This process protects surrounding cells and contributes to a decrease in overall oxidative damage [25]. Studies investigating the antioxidant properties of PRP, found that PRP treatment was able to relieve the accumulation of ROS and the degree of lipid oxidation by upregulating SOD, GSH-Px, and CAT activity levels [26–29]. By reducing oxidative stress, PRP can protect sperm cells from ROS-induced damage, thereby improving their functional integrity.

The harmful effects of ROS extend beyond immediate sperm function; they can also lead to apoptosis and impair the fertilization process. Elevated ROS levels can disrupt mitochondrial function, which is vital for sperm motility, and initiate pathways that result in cell death [30]. Indeed, mitochondria are a significant source of ROS during ATP production via oxidative phosphorylation. Our findings revealed that PRP supplementation is associated with improved mitochondrial function, as evidenced by enhanced activity of mitochondrial enzymes such as SDH. SDH is a crucial enzyme involved in both the citric acid cycle (Krebs cycle) and the mitochondrial electron transport chain (complex II). Therefore, our findings demonstrated an improvement in both energy production via the Krebs cycle and mitochondrial chain function. Similarly to sperm oxidative stress reduction, PRP at 5% present the best improvement in mitochondria function (80%, p = 0.002). Our results are in line with a recent study, which demonstrated that PRP contributes in repairing the mitochondrial function by activating the AMPK/NF-κB signaling pathway in osteoarthritic chondrocytes [31]. Another study showed that PRP preserve the maintain of mitochondrial activity of maturing oocytes during *in vitro* oocyte maturation [32].

PRP concentration is critical in determining its effects on spermatozoa. When testing 2%, 5% and 10% PRP, we have demonstrated a beneficial impact of the three studied concentrations among which 5% concentration displays superior improvements in both ROS reducing and SDH activity enhancement. This outcome might be due to the optimal concentration of growth factors and cytokines that enhance cellular functions without overwhelming the spermatozoa. The 5% concentration likely provides an ideal balance of these components. The lower concentration 2%, while beneficial, may not provide the full spectrum of protective effects seen with 5% PRP. Conversely, higher concentrations like 10% may lead to saturation or even adverse effects, potentially due to increased viscosity or other factors that could hinder sperm function.

Although the findings of the current study provide new insights into an underexplored field of the use of PRP that is male infertility through highlighting the beneficial impact of PRP supplementation in improving semen oxidative status in infertile patients, it bears mentioning that it has some limitations mainly related to the use of only two tests (MTT and NBT assays). It could be of great interest to perform further studies investigating lipid peroxidation and DNA sperm integrity.

## Conclusion

In conclusion, our findings underscore PRP’s potential in mitigating sperm oxidative stress and enhancing mitochondrial function, which are pivotal elements in preserving sperm quality. Given the promising results of *our in vitro* study, it could be an interesting start point for future clinical trials assessing the effectiveness of PRP during ART procedures in order to optimize take home baby rate.

## Supporting information

S1 Table Data. (XLSX)

## Abbreviations

ART: assisted reproductive technologies
PRP: platelet rich plasma
NBT: nitroblue tetrazolium
MTT: mitochondrial membrane potential
DNA: desoxy ribonucleic acid
TGF b: growth factor b
FGF: fibroblast growth factor
VEGF: vascular endothelial growth factor
IGF-1: insulin-like growth factor 1
PDGF: platelet-derived growth factor
Zn: zinc
SDH: succinate dehydrogenase
SOD: superoxide dismutase
IVF: in vitro fertilization
OS: oxidative stress.
